# Age and body weight-adjusted infusion rate (mg/kg/h) as risk factors for vancomycin infusion reaction in patients receiving perioperative antimicrobial prophylaxis: a retrospective cohort study

**DOI:** 10.1093/jacamr/dlag084

**Published:** 2026-05-19

**Authors:** Osamu Iketani, Shunsuke Uno, Masayoshi Shinjoh, Ryo Takemura, Shogo Fukuda, Shoji Seyama, Motonori Kimura, Haruki Ishikawa, Tomohisa Hayakawa, Yoshifumi Uwamino, Ho Namkoong, Ayumi Yoshifuji, Hideaki Obara, Yaoko Takano, Yuki Enoki, Kazuaki Taguchi, Yasunori Sato, Kazuaki Matsumoto, Naoki Hasegawa

**Affiliations:** Division of Infectious Diseases and Infection Control, Keio University Hospital, Tokyo, Japan; Division of Infectious Diseases and Infection Control, Keio University Hospital, Tokyo, Japan; Department of Infectious Diseases, Keio University School of Medicine, Tokyo, Japan; Division of Infectious Diseases and Infection Control, Keio University Hospital, Tokyo, Japan; Department of Pediatrics, Keio University School of Medicine, Tokyo, Japan; Department of Biostatistics, Keio University School of Medicine, Tokyo, Japan; Division of Infectious Diseases and Infection Control, Keio University Hospital, Tokyo, Japan; Division of Infectious Diseases and Infection Control, Keio University Hospital, Tokyo, Japan; Department of Pharmacy, Keio University Hospital, Tokyo, Japan; Department of Clinical Microbiology, School of Pharmacy, Tokyo University of Pharmacy and Life Sciences, Tokyo, Japan; Department of Pharmacy, Keio University Hospital, Tokyo, Japan; Department of Pharmacy, Keio University Hospital, Tokyo, Japan; Department of Pharmacy, Keio University Hospital, Tokyo, Japan; Division of Infectious Diseases and Infection Control, Keio University Hospital, Tokyo, Japan; Department of Laboratory Medicine, Keio University School of Medicine, Tokyo, Japan; Division of Infectious Diseases and Infection Control, Keio University Hospital, Tokyo, Japan; Department of Infectious Diseases, Keio University School of Medicine, Tokyo, Japan; Division of Infectious Diseases and Infection Control, Keio University Hospital, Tokyo, Japan; Department of Infectious Diseases, Keio University School of Medicine, Tokyo, Japan; Division of Infectious Diseases and Infection Control, Keio University Hospital, Tokyo, Japan; Department of Surgery, Keio University School of Medicine, Tokyo, Japan; Division of Infectious Diseases and Infection Control, Keio University Hospital, Tokyo, Japan; Division of Pharmacodynamics, Keio University Faculty of Pharmacy, Tokyo, Japan; Division of Pharmacodynamics, Keio University Faculty of Pharmacy, Tokyo, Japan; Department of Biostatistics, Keio University School of Medicine, Tokyo, Japan; Division of Pharmacodynamics, Keio University Faculty of Pharmacy, Tokyo, Japan; Division of Infectious Diseases and Infection Control, Keio University Hospital, Tokyo, Japan; Department of Infectious Diseases, Keio University School of Medicine, Tokyo, Japan

## Abstract

**Background:**

Vancomycin infusion reaction (VIR) is a common adverse reaction to vancomycin. We aimed to investigate the effects of infusion rate, age, infusion time, renal function and history of allergy on the incidence of VIR.

**Methods:**

We conducted a single-centre retrospective cohort study with 153 patients (aged 0–92.7 years) who received a single intravenous dose of vancomycin for perioperative prophylaxis. Patients treated for active infections were excluded. Medical records were retrospectively evaluated for VIR occurrence, defined as itching, flushing or rash documentation.

**Results:**

VIR incidence was 46.7% (7/15) in patients aged <10 years, 47.4% (9/19) in those aged 10–20 years and 44.4% (4/9) in those aged 20–30 years, versus 2.7% (2/74) in those aged ≥60 years. Multivariable logistic regression identified younger age [adjusted odds ratio (aOR) 0.95, 95% CI 0.93–0.97] and higher weight-adjusted infusion rate (aOR 1.22 per mg/kg/h increase, 95% CI 1.04–1.43) as independent risk factors for VIR. Receiver operating characteristic analysis of the infusion rate by age group showed that the optimal cut-off value for patients aged 30–60 years was 14.8 mg/kg/h, whereas for those aged <30 years, it was 14.0 mg/kg/h. No patients developed VIR when the infusion rate was <9.4 mg/kg/h.

**Conclusions:**

Age and higher weight-adjusted infusion rate were key risk factors for VIR. Tailoring infusion rate to age and weight may reduce VIR incidence without unnecessarily prolonging administration time.

## Introduction

Vancomycin has a histamine-releasing effect,^1–5^ leading to the characteristic vancomycin infusion reaction (VIR) side effect,^6–8^ which can, in some cases, make it challenging to continue vancomycin therapy.^9^ Previously termed ‘red man syndrome’ or ‘red neck syndrome’, VIR is now more accurately referred to as VIR.^10–12^

The reported VIR incidence varies widely in the literature, ranging from 3.4% to 50%,^13–16^ with evidence suggesting that the infusion time of vancomycin at the time of administration may influence its occurrence.^7,17^ Rapid vancomycin administration (e.g. within 10 min) could precipitate VIR. A rapid infusion rate of 1000 mg/h has been reported to cause a high VIR incidence (81.8%) in healthy volunteers, which is significantly higher than the incidence at a slower rate of 500 mg/h. Additionally, the vancomycin infusion rate reportedly correlates with both serum histamine levels and VIR severity.^3^ As a preventive measure, vancomycin is now generally administered over at least 1 h. Various alternative administration methods have also been recommended, such as a maximum infusion rate of 10 mg/min, maintaining a final concentration of ≤5 mg/mL and infusing for at least 30 min per 500-mg dose.^2,3,17–19^ Conversely, some patients develop VIR even with infusions lasting ≥2 h, suggesting that factors other than the infusion rate may contribute to the risk.^2^

Age has also been identified as a risk factor for VIR. A particularly high VIR incidence has been reported in paediatric patients, especially those aged 2 years and older.^20^ An elevated incidence of VIR has been observed in patients aged <40 years, indicating that age may be a significant risk factor for VIR in those aged ≥16 years.^13^

During a national shortage of cephalosporin antibiotics, such as cefazolin, in Japan, Keio University Hospital used vancomycin as a perioperative antibiotic per domestic and international guideline recommendations.^21–25^ Despite administering vancomycin over at least 1 h to mitigate VIR risk, several cases of VIR were observed in paediatric patients.

This was a retrospective cohort study analysing pooled data to examine the association between VIR onset and several variables, including infusion time, age, infusion rate, renal function and history of allergy. Through statistical analysis, we identified the factors associated with VIR onset.

## Methods

### Patient selection

We included adult and paediatric patients who received vancomycin as perioperative prophylactic antibiotics between March 18 and March 29, 2019, when there were supply restrictions on cefazolin. Patients who received a single dose of vancomycin prior to surgery at Keio University Hospital (32 departments, 950 beds, Tokyo, Japan) were identified from the electronic medical record system, and patients who received vancomycin for the treatment of an infection were excluded. Patients were categorized into two groups based on the development of VIR (VIR-positive and VIR-negative). Each patient was included only once in the analysis (their first surgical event during the study period was considered), to ensure independent observations. Throughout the investigation period, our hospital recommended the administration of vancomycin at a dose of 15 mg/kg infused over a minimum of 1 h, in compliance with Japanese national guidelines for perioperative antimicrobial prophylaxis and the prescribing information.^26,27^ We excluded patients who underwent concurrent administration of vancomycin and other agents that cause histamine-releasing effects and VIR. These agents included ciprofloxacin, amphotericin B, rifampicin, teicoplanin, opioid analgesics, muscle relaxants, contrast media, barbiturates, propofol and plasma.^28,29^ Additionally, events that the attending physician attributed to medical factors other than vancomycin were excluded from the analysis.

The study was conducted in accordance with the Code of Ethics of the World Medical Association (Declaration of Helsinki) and was approved by the Keio University School of Medicine Ethics Committee (approval number 20140032). An opt-out procedure was implemented, whereby information about the study was made publicly available on the institutional website, allowing patients to decline participation if they wished.

### Patient background

Patient demographic and clinical characteristics—including age, weight, allergy history, VCM administration details, renal function parameters and concomitant medications—were extracted from medical records, as detailed in Table 1.Underlying diseases were grouped into nine categories based on the major diagnostic categories of the International Classification of Diseases, 10th Revision to ensure a standardized classification: (i) certain infectious and parasitic diseases; (ii) congenital malformations, deformations and chromosomal abnormalities; (iii) circulatory system diseases; (iv) digestive system diseases; (v) genitourinary system diseases; (vi) musculoskeletal and connective tissue diseases; (vii) nervous system diseases; (viii) endocrine, nutritional and metabolic diseases and (ix) neoplasms.

**Table 1.** Risk factors for patients with or without vancomycin infusion reaction

Characteristics	No vancomycin infusion reaction (*n* = 126)	Vancomycin infusion reaction (*n* = 27)	*P*-value
Age, median (IQR)	65.75 (43.6–75.2)	15.3 (9.1–39.4)	<0.0001
Sex, male (%)	59 (46.83)	10 (37.04)	0.3995
Body weight (kg), mean (SD)	56.18 (18.22)	40.73 (14.88)	<0.0001
Height (cm), mean (SD)	155.89 (22.16)	145.29 (22.3)	0.0258
BMI, mean (SD)	22.41 (4.91)	18.45 (2.83)	<0.0001
BSA (m2), mean (SD)	1.54 (0.34)	1.28 (0.34)	0.0004
Food or drug allergy, *n* (%)	54 (42.86)	14 (51.85)	0.4031
Vancomycin dose, (mg/dose), median (IQR)	900 (600–1000)	650 (440–900)	0.0020
Infusion time (min), median (IQR)	60 (60–60)	60 (60–60)	0.1687
vancomycin concentration (mg/mL), median (IQR)	9 (7.5–10)	6.5 (4.5–9)	0.0002
Administration rate 1 (mg/min), median (IQR)	15.0 (8.3–16.7)	10.8 (7.3–15.0)	0.0141
Administration rate 2 (mg/kg/h), median (IQR)	14.3 (12.5–16.0)	15.2 (14.1–16.0)	0.0189
Serum creatinine (mg/dL), median (IQR)	0.7 (0.58–0.87)	0.58 (0.46–0.72)	0.0061
BUN (mg/dL), median (IQR)	14.3 (11.1–18)	12.3 (9.6–14.1)	0.0756
e-GFR (mL/min/1.73 m2), median (IQR)	72 (64–83.5)	73 (61–107)	0.4783
Concomitant medications use			
H1 receptor antagonist, *n* (%)	12 (9.52)	2 (7.41)	1
H2 receptor antagonist, *n* (%)	10 (7.94)	3 (11.11)	0.7021
Corticosteroid (systemic), *n* (%)	12 (9.52)	5 (18.52)	0.1848
Tracheostomy, *n* (%)	1 (0.79)	0 (0)	
Underlying disease			<0.0001
Certain infectious and parasitic diseases, *n* (%)	1 (0.79)	0 (0)	
Congenital malformations, deformations and chromosomal abnormalities, *n* (%)	2 (1.59)	5 (18.52)	
Diseases of the circulatory system, *n* (%)	17 (13.49)	4 (14.81)	
Diseases of the digestive system, *n* (%)	13 (10.32)	1 (3.7)	
Diseases of the genitourinary system, *n* (%)	2 (1.59)	0 (0)	
Diseases of the musculoskeletal system and connective tissue, *n* (%)	44 (34.92)	11 (40.74)	
Diseases of the nervous system, *n* (%)	3 (2.38)	0 (0)	
Endocrine, nutritional and metabolic diseases, *n* (%)	1 (0.79)	0 (0)	
Neoplasms, *n* (%)	43 (34.13)	6 (22.22)	

### VIR definition

The definition of VIR was established according to previous reports.^4,8,9,14,21,29^ A patient was considered to have VIR if they exhibited one or more of the following symptoms: itching, redness/flushing or skin rash on the head, face, neck, anterior chest or back. Symptoms reported by patients or their families were subsequently confirmed by healthcare professionals and investigated through a review of medical records. Symptom onset time was established as the earliest documented occurrence, whether from a patient report or professional confirmation.

### Statistical analysis

An *a priori* power analysis was not conducted because the sample size was strictly determined by the total number of eligible patients who received vancomycin during the brief timeframe of a national cefazolin shortage. Descriptive statistics are presented as counts and proportions for categorical variables, and as means ± standard deviations or medians with ranges for continuous variables, depending on their distribution. For group comparisons, a *t*-test was employed for continuous variables. Categorical variables were analysed using the chi-square test or Fisher's exact test, as appropriate. Significant risk factors for adverse reactions identified in univariate analyses were incorporated into a multivariable logistic regression model, utilizing a stepwise selection method with entry and removal thresholds set at *P*-values of 0.05. The predictive accuracy of the model was assessed using the area under the receiver operating characteristic curve (AUC). Comparisons between Receiver operating characteristic (ROC) curves were conducted using the DeLong test for correlated ROC curves. ROC analysis was conducted for the weight-adjusted infusion rate. The age strata used for the stratified ROC analysis (<30, 30–60 and >60 years) were determined *post hoc* based on the observed distribution of VIR incidence across the age groups.

Items with missing data were handled using a complete-case analysis, where data points with missing values were excluded from the statistical analysis. In the multinomial logistic regression analysis, variables with variance inflation factors exceeding acceptable limits were excluded to mitigate multicollinearity. All statistical analyses were performed using SPSS version 21 (IBM Corp., Armonk, NY, USA) and JMP Pro 17 (SAS Institute Inc., Cary, NC, USA). A *P*-value of <0.05 was considered statistically significant.

## Results

During the study period, vancomycin was administered preoperatively to 153 patients (Table 1). A review of the medical records revealed that none of the patients received any of the excluded concomitant medications at the time of vancomycin administration. Among these patients, 27 developed VIR, resulting in an incidence of 17.6%. The median [interquartile range (IQR)] age of the VIR-positive group was 15.3 (9.1–39.4) years, while the median age of the VIR-negative group was 65.75 (43.6–75.2) years (*P* < 0.0001). Additionally, the VIR-positive group exhibited lower body weight (kg) (*P* < 0.0001), height (*P* = 0.0258), body mass index (*P* < 0.0001) and body surface area (m^2^) (*P* = 0.0004) compared to the VIR-negative group. No differences were found between the groups regarding sex, use of H1-blockers, H2-blockers, steroids or history of food or drug allergies. The underlying diseases varied between the two groups (*P* < 0.0001) (Table 1).

Vancomycin was generally administered over a period of at least 1 h in the hospital, except for one patient who received an infusion lasting 50 min. The median infusion time was 60 (60–60) min for both the VIR-positive and VIR-negative groups, with both groups averaging >1 h. The median infusion rate (IQR) was 10.8 (7.3–15) mg/min in the VIR-positive group and 15 (8.3–16.7) mg/min in the VIR-negative group, with both median infusion rates exceeding the recommended 10 mg/min (*P* = 0.0141). We found that 27.8% (35/126) of patients who did not develop VIR received the infusion at the recommended rate of ≤ 10 mg/min, compared with 37.0% (10/27) of patients who did develop VIR. The median concentration of vancomycin (IQR) was slightly lower in the VIR-positive group at 6.5 (4.5–9) mg/mL compared to 9 (7.5–10) mg/mL in the VIR-negative group (*P* = 0.0002). The median weight-adjusted infusion rate was higher in the VIR-positive group [15.2 (14.1–16) mg/kg/h] compared with the VIR-negative group [14.3 (12.5–16) mg/kg/h, *P* = 0.0189]. The median (IQR) serum creatinine levels were 0.58 (0.46–0.72) in the VIR-positive group and 0.70 (0.58–0.87) in the VIR-negative group (*P* = 0.0061), with both groups exceeding 1 h on average. No differences were observed between the two groups regarding BUN (*P* = 0.0756) and eGFR (*P* = 0.4783), which relate to renal function (Table 1).

Details of all patients who developed VIR are presented in Table 2. The most common symptom was pruritus, observed in 22 patients (81.5%), followed by flushing in 18 patients (66.7%) and rash in three patients (11.1%). The infusion rate varied from 2.5 mg/min to 16.7 mg/min, with lower rates in younger patients; the minimum weight-adjusted infusion rate was 9.4 mg/kg/h (Table 2).

**Table 2.** Patient characteristics of all patients with vancomycin infusion reaction

	Sex (Male or Female)	Age (years)	Body weight (kg)	Food or Drug Allergy	Pruritus	Flush	Rash	H1 receptor antagonist	H2 receptor antagonist	Steroid (systemic)	Onset time (min)	vancomycin dose (mg/dose)	Diluent volume (mL)	Administration time (min)	vancomycin conc. (mg/mL)	Administration rate 1 (mg/min)	Administration rate 2 (mg/kg/h)
No.1	F	1.9	10.5	+	+		+				58	150	100	60	1.5	2.5	14.3
No.2	F	4.5	21.5		+		+				60	280	56	60	5	4.7	14.7
No.3	F	6.3	18.7	+	+						37	300	100	60	3	5.0	16.0
No.4	F	7.9	26.2	+	+	+					90	370	100	90	3.7	4.1	9.4
No.5	M	8.9	28.4		+	+					60	420	84	60	5	7.0	14.8
No.6	F	9.1	28			+				+	30	440	100	90	4.4	7.3	15.7
No.7	M	9.1	25	+	+						60	340	100	60	3.4	5.7	13.6
No.8	M	10.6	28.8		+	+					30	440	100	60	4.4	7.3	15.3
No.9	M	10.7	30.6		+	+					40	500	100	50	5	10.0	19.6
No.10	F	12.5	26.6			+		+	+	+	13	450	100	60	4.5	7.5	16.9
No.11	M	12.6	53.4		+	+					55	750	100	60	7.5	12.5	14.0
No.12	F	13.1	35.9		+	+				+	43	450	100	60	4.5	7.5	12.5
No.13	F	14.2	52		+	+					55	750	100	60	7.5	12.5	14.4
No.14	F	15.3	45.1	+	+	+					30	700	100	60	7	11.7	15.5
No.15	F	16.6	42.7	+	+	+					60	600	100	60	6	10.0	14.1
No.16	M	17	45.1	+		+					20	1000	100	60	10	16.7	22.2
No.17	F	21.4	49.2		+	+			+		45	720	100	60	7.2	12.0	14.6
No.18	F	21.4	51	+		+		+	+	+	60	720	72	60	10	12.0	14.1
No.19	F	23.4	42.7	+	+						45	650	100	60	6.5	10.8	15.2
No.20	M	25.3	59.9		+						60	900	100	60	9	15.0	15.0
No.21	F	39.4	60.05		+	+					30	1000	100	60	10	16.7	16.7
No.22	F	44.4	56.6	+		+					—	900	100	60	9	15.0	15.9
No.23	M	46.8	55.2	+	+						60	1000	100	60	10	16.7	18.1
No.24	M	48.5	49	+	+	+	+			+	45	1000	100	60	10	16.7	20.4
No.25	F	58	56.3	+	+						60	900	100	60	9	15.0	16.0
No.26	F	71.2	38.2	+	+	+					60	600	100	60	6	10.0	15.7
No.27	M	79.2	65.5		+						60	900	100	60	9	15.0	13.7

**Figure 1.**
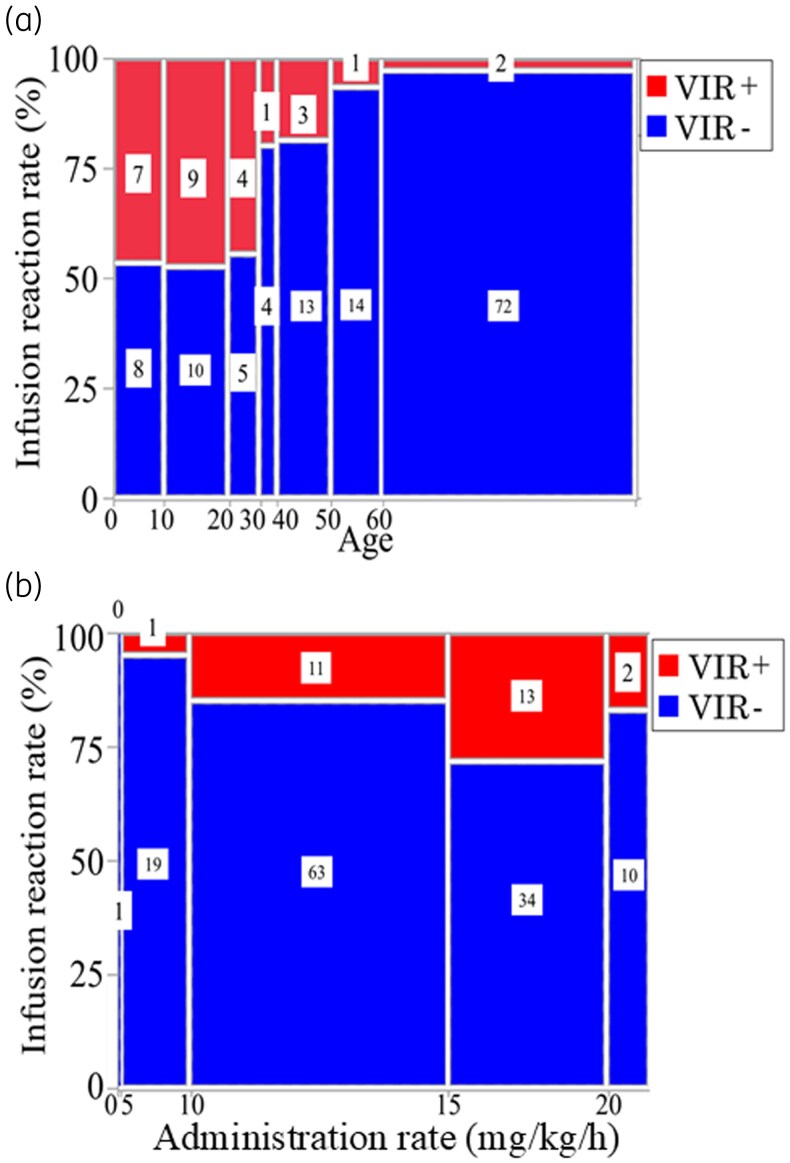
(a) Mosaic plot of vancomycin infusion reaction for each age group (years), number of patients and proportion (%). (b) Mosaic plot of vancomycin infusion reaction based on the administration rate (mg/kg/h), with the number of patients and proportion (%) shown.

The mosaic plot of the proportion of VIR by age is shown in Figure 1a. VIR incidence was notably higher in younger age groups, with rates of 46.7% (7/15) in patients aged <10 years, 47.4% (9/19) in those aged 10–20 years and 44.4% (4/9) in those aged 20–30 years. In patients aged 30–40 years, the incidence was 20.0% (1/5), while in the 40–50-year group, it was 18.8% (3/16). The incidence further decreased to 6.7% (1/15) in the 50–60-year age group and was lower in individuals aged >60 years at 2.7% (Table 1). VIR onset occurred between 13 and 90 min after the end of the infusion, most commonly occurring near the end of the infusion.

Figure 1b illustrates a mosaic plot of the proportion of VIR for each infusion rate adjusted for body weight (mg/kg/h). The incidence was 0% (0/1) for rates <5 mg/kg/h, 5.0% (1/20) for rates of 5–10 mg/kg/h, 14.9% (11/74) for rates of 10–15 mg/kg/h, 27.7% (13/47) for rates of 15–20 mg/kg/h and 16.7% (2/12) for rates >20 mg/kg/h. VIR incidence tended to increase with higher weight-adjusted infusion rates.

A significant difference was observed between the groups in the univariate analysis. A multivariable logistic regression analysis was performed, including age and several potential risk factors for VIR development: (i) vancomycin infusion time (min), (ii) infusion rate (mg/min) and (iii) weight-adjusted infusion rate (mg/kg/h). Although age differed across all groups (*P* < 0.0001), there was no association found between infusion time [adjusted odds ratio (aOR): 0.96, 95% confidence interval (CI): 0.90–1.03, *P* = 0.265] or infusion rate (aOR: 1.06, 95% CI: 0.95–1.19, *P* = 0.2774). However, the weight-adjusted infusion rate was associated with VIR development, with an aOR of 1.22 (95% CI: 1.04–1.43, *P* = 0.0131) (Table 3).

**Table 3.** Multivariate risk factor for vancomycin infusion reaction

Model	Factors	Adjusted OR (95%CI)	*P* value
Model 1	Age (years)	0.95 (0.93–0.97)	<0.0001
	infusion time (min)	0.96 (0.90–1.03)	0.265
Model 2	Age (years)	0.94 (0.92–0.97)	<0.0001
	Administration rate (mg/min)	1.06 (0.95–1.19)	0.2774
Model 3	Age (years)	0.95 (0.93–0.97)	<0.0001
	Administration rate (mg/kg/h)	1.22 (1.04–1.43)	0.0131

**Figure 2.**
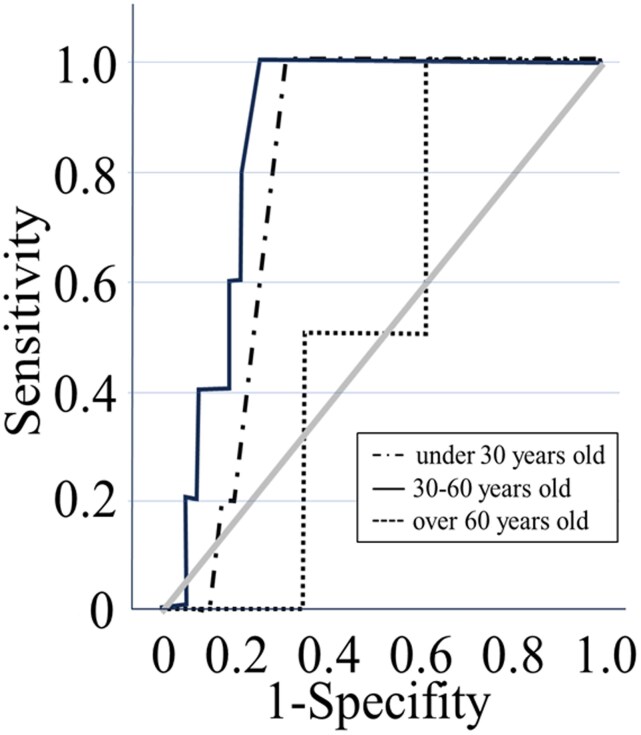
ROC curve analysis of vancomycin infusion reaction predictors based on infusion rate adjusted body weight (mg/kg/h). Categories include patients aged <30 years (AUC = 0.69, *P* < 0.05), 30–60 years (AUC = 0.86, *P* < 0.01) and >60 years (AUC = 0.54, *P* = 0.718).

ROC analysis was conducted for the weight-adjusted infusion rate. The age strata (<30, 30–60 and >60 years) used for the stratified ROC analysis were determined *post hoc* based on the observed distribution of VIR incidence across the age groups. The ROC curve indicated that the weight-adjusted infusion rate was not predictive of VIR in patients aged >60 years (AUC: 0.54, 95% CI: 0.33–0.75, *P* = 0.718). In contrast, in the 30–60-year cohort, the AUC was 0.86 (95% CI: 0.74–0.98, *P* < 0.01), indicating a strong association with the weight-adjusted infusion rate. For patients aged <30 years, the AUC was 0.69 (95% CI: 0.52–0.85, *P* < 0.05), suggesting a moderate association (Figure 2). Using the Youden index, cut-off values were determined from the ROC curve: 14.8 mg/kg/h for patients aged 30–60 years and 14.0 mg/kg/h for those aged <30 years.

## Discussion

In this study, we investigated the risk factors for VIR in patients who received a single dose of vancomycin for perioperative prophylaxis. Our findings indicate that age and the infusion rate adjusted for body weight (mg/kg/h) influenced VIR incidence.

The median age of the patients who developed VIR was 15.3 years, which was lower than that of the patients who did not develop VIR (65.7 years), suggesting that age is a risk factor for VIR. Furthermore, VIR incidence was notably high, at approximately 50%, for patients aged <30 years. This incidence decreased to approximately 20% for those aged between 30 and 50 years, and for individuals aged ≥60 years, the incidence was only 2.7%. This trend indicates a strong age-related effect on the likelihood of developing VIR.

In this study, the overall VIR incidence was 17.6% (27/153). While this rate is low, the incidence among patients <20 years old was 47.1% (16/34), which is higher than in previously published studies. In our study, the most important factor contributing to the high VIR incidence was the difference in the study population, particularly whether vancomycin was administered to patients with or without infections. Healy *et al*.^13^ reported a VIR incidence rate of 80% (8/10) in a study involving healthy young adults (average age: 24.6 years) who received a 1-h intravenous infusion of 1 g of vancomycin. Similarly, in a study by Sahai *et al*.^30^ involving healthy adults (mean age: 24.1 years), the VIR incidence rate during vancomycin administration (15 mg/kg over 60 min) was reported 91.7% (11/12). Rybak *et al*. and other reports suggest that factors contributing to the higher VIR incidence, compared to its use in infection treatment, include underlying conditions in hospitalized patients (e.g. febrile and septic patients and malignant tumours) and concomitant medications, which may alter the normal release or response to histamine. In this study, we focused on patients receiving preoperative antibiotics, rather than those being treated for infections, and included patients with minimal histamine secretion related to infection and minimal influence of steroids or/antihistamines. Thus, the VIR incidence rate was similar to that observed in healthy individuals.

Myers *et al*. reported an incidence of 14.1% (77/546) for VIR in patients <21 years old, which was lower than the incidence in our patient cohort (<20 years old; 47.1%, 16/34). Myers and colleagues asserted that their criteria for determining whether a subject developed VIR were more stringent than previously established clinical criteria, suggesting the possibility that they may have excluded subjects with milder symptomatology. They defined VIR as a patient with at least two symptoms. Thus, the difference in incidence between their and our studies could be attributed to their exclusion of patients with only one symptom. If we apply the same definition, the incidence among patients <20 years of age would decrease to 35.3% (12/34). Moreover, 7 of the 27 patients (25.9%) who developed VIR reported subjective itching without objective symptoms. This incidence was slightly higher than that reported by Korman *et al*., who described an association between VIR and age (2/12 patients, 16.7%). Since all of these patients’ symptoms resolved immediately after the infusion was discontinued, it is possible that our study included very mild cases.

We observed no significant differences between the two groups regarding the use of antihistamines or systemic steroids, despite ongoing controversy among clinicians about the relationship between VIR and histamine levels, as well as the preventive effect of antihistamines.^2,4,5,15,16,31,32^ In our study, the combined effects of these treatments did not show impact.

Apart from age, the most commonly reported risk factor for VIR since its initial documentation in 1985 is an infusion time of <1 h.^3,6,7,12,17,31,33^

The Infectious Diseases Society of America guidelines for antimicrobial prophylaxis recommend a dose of 15 mg/kg administered over 1 to 2 h. However, the 2020 Vancomycin TDM Consensus Guidelines recommend an administration time of over 60 min and a rate of no more than 10 mg/min. Nevertheless, Levy *et al*.^14^ reported cases of VIR occurring at rates of 10 mg/min or less. Furthermore, Healy *et al*.^2^ reported that 30% (3/10) of patients developed VIR at doses ≤10 mg/min. Therefore, we recommend administering 15 mg/kg over 60 min as the most convenient and easily standardized method for determining perioperative prophylactic antibiotic doses within our institution. However, this approach raises concerns about the risk of VIR, as the increased dosage might result in an infusion rate greater than 10 mg/min. Interestingly, our data yielded some surprising results. The median infusion rate for patients who developed VIR was 10.8 mg/min, which is consistent with the recommended guidelines. Contrastingly, the median infusion rate for patients who did not develop VIR was 15.0 mg/min, which was higher.

Additionally, both the concentration (mg/mL) and infusion rate (mg/min), which are considered risk factors, were lower in the VIR group than in the non-VIR group. Notably, many children with low body weight developed VIR, even when the infusion rate was below the recommended threshold of 10 mg/min. This suggests that the weight-adjusted infusion rate (mg/kg/h) may be more critical than the infusion rate per patient (mg/min) in preventing VIR (Table 1).

In this study, we conducted a multivariate analysis that included age, infusion time (min), administration rate (mg/min) and the weight-adjusted infusion rate (mg/kg/h). The results demonstrated that only age and the weight-adjusted infusion rate (mg/kg/h) were correlated with VIR development (Table 3). Current guidelines do not mention age or weight-adjusted infusion rate as risk factors for VIR, and specifically, whether the infusion rate for patients <60 years of age should be lower than the weight-adjusted infusion rate for patients >60 years of age.

When comparing the ROC curve for VIR incidence with the infusion rate adjusted for body weight, VIR incidence was not affected by the infusion rate in patients aged ≥60 years. However, it was most influenced by the weight-adjusted infusion rate in patients aged 30–60 years, with a cut-off value of 14.8 mg/kg/h. VIR incidence observed in patients who received the recommended infusion method, and the cut-off value obtained in this study are presented in Table 4. Notably, when the infusion was administered at the cut-off value of 14.8 mg/kg/h, VIR incidence was reduced to 0%. Conversely, for patients aged <30 years—who represent the highest risk group for developing VIR—the incidence was lowered to 18.8% when vancomycin was administered at the cut-off value of 14.0 mg/kg/h, compared to 45.2% when the infusion time of <1 h was used (see Table 4 for a comprehensive comparison). Furthermore, no patients developed VIR when the dose was ≤9.4 mg/kg/h. For young patients under 30 years of age who need to be cautious about developing VIR, it may be advisable to consider administering a dose of ≤9.4 mg/kg/h as a preventive strategy, similar to that recommended for high-risk patients with a prior history of VIR.

**Table 4.** Incidence of vancomycin infusion reaction for each vancomycin administration rate

Age	infusion time > 60 min	Administration rate 10 mg/min	Recommended rate* (mg/kg/h)
30 years > Age	45.2% (19/42)	45.5% (10/22)	18.8% (3/16)
Age: 30–60 years	13.9% (5/36)	0% (0/5)	0% (0/21)
60 years < Age	2.7% (2/74)	0% (0/18)	—
Total	17.1% (26/152)	22.2% (10/45)	

* Recommended rate was defined as 14.0 mg/kg/h for patients aged <30 years and 14.8 mg/kg/h for patients aged 30–60 years, based on ROC curve analysis; no recommended rate was defined for patients aged >60 years.

In 2019, a severe shortage of cefazolin in Japan necessitated the temporary use of vancomycin as a perioperative prophylactic antimicrobial agent until alternative drugs became available. This timeframe encompassed surgeries performed in hospitals, resulting in a patient cohort with a wider age range than typically seen in infectious disease treatment. Additionally, it is possible that the risk factors for VIR were assessed under conditions that were less influenced by concomitant medications, underlying diseases and complications than those more common in patients receiving treatment for infections. We believe that these data hold significant value for informing treatment decisions, and such a comprehensive comparison may be challenging to replicate in the future.

The limitations of this study include its focus solely on the period when vancomycin was employed as a perioperative antibiotic due to a nationwide shortage of cephalosporin antibiotics, such as cefazolin, and the relatively short duration of the study. Additionally, the number of patients in each age group was limited. As an *a priori* power calculation was not performed, our study risks being underpowered. The overall number of patients who developed VIR was low, which introduces the possibility of Type II errors; furthermore, other potential risk factors evaluated may have been under-represented and therefore gone undetected in our population. Furthermore, the study exclusively evaluated the perioperative administration of antibiotics, leaving it unclear whether similar results would be observed in patients undergoing treatment for infections. Future research should aim to expand the scope of the study to include patients being treated for infections, alongside those receiving perioperative antibiotic therapy, and to examine a larger patient population to enhance the generalizability of our conclusions.

Over the years, numerous reports have addressed the risk factors and preventive measures for VIR. This study has revealed that both age and the weight-adjusted infusion rate (mg/kg/h) are risk factors for VIR, with age exerting a particularly strong influence. Our findings suggest that age-specific, weight-adjusted infusion rates may serve as an indicator for effectively preventing VIR without necessitating unnecessarily prolonged administration, thereby offering a viable strategy for minimizing VIR incidence while reducing the burden on patients.

In conclusion, our retrospective study suggests that VIR incidence is associated with both patient age and the weight-adjusted administration rate. We found that that VIR incidence is lower in older patients, so a simple infusion time of ≥1 h may be sufficient for this population. Conversely, a higher incidence of VIR was observed in younger patients, for whom weight-adjusted administration rates appear critical in risk reduction. Further prospective studies with larger patient cohorts are needed to validate these findings and establish specific administration guidelines.

## References

[1] Rybak MJ, Le J, Lodise TP et al Therapeutic monitoring of vancomycin for serious methicillin-resistant Staphylococcus aureus infections: a revised consensus guideline and review by the American Society of Health-System Pharmacists, the Infectious Diseases Society of America, the Pediatric Infectious Diseases Society, and the Society of Infectious Diseases Pharmacists. Clin Infect Dis 2020; 71: 1361–4. 10.1093/cid/ciaa303 32658968

[2] Healy DP, Sahai JV, Fuller SH et al Vancomycin-induced histamine release and “red man syndrome”: comparison of 1- and 2-hour infusions. Antimicrob Agents Chemother 1990; 34: 550–4. 10.1128/AAC.34.4.550 1693055 PMC171642

[3] Polk RE, Healy DP, Schwartz LB et al Vancomycin and the red-man syndrome: pharmacodynamics of histamine release. J Infect Dis 1988; 157: 502–7. 10.1093/infdis/157.3.502 2449506

[4] Renz CL, Thurn JD, Finn HA et al Oral antihistamines reduce the side effects from rapid vancomycin infusion. Anesth Analg 1998; 87: 681–5. 10.1213/00000539-199809000-00036 9728853

[5] Renz CL, Thurn JD, Finn HA et al Antihistamine prophylaxis permits rapid vancomycin infusion. Crit Care Med 1999; 27: 1732–7. 10.1097/00003246-199909000-00006 10507591

[6] Bailie GR, Yu R, Morton R et al Vancomycin, red neck syndrome, and fits. Lancet 1985; 2: 279–80. 10.1016/S0140-6736(85)90332-0 2862455

[7] Garrelts JC, Peterie JD. Vancomycin and the “red man’s syndrome”. N Engl J Med 1985; 312: 245. 10.1056/NEJM198501243120416 3155563

[8] Holliman R . Red man syndrome’ associated with rapid vancomycin infusion. Lancet 1985; 1: 1399–400. 10.1016/S0140-6736(85)91831-8 2861353

[9] Cole DR, Oliver M, Coward RA et al Allergy, red man syndrome, and vancomycin. Lancet 1985; 2: 280. 10.1016/S0140-6736(85)90333-2 2862456

[10] Rothenberg HJ . Anaphylactoid reaction to vancomycin. JAMA 1959; 171: 1101–2. 10.1001/jama.1959.73010260008010b 14439446

[11] Alvarez-Arango S, Ogunwole SM, Sequist TD et al Vancomycin infusion reaction – moving beyond “red man syndrome”. N Engl J Med 2021; 384: 1283–6. 10.1056/NEJMp2031891 33830710 PMC9235042

[12] Austin JP, Foster BA, Empey A. Replace red man syndrome with vancomycin flushing reaction. Hosp Pediatr 2020; 10: 623–4. 10.1542/hpeds.2020-0125 32571794

[13] Korman TM, Turnidge JD, Grayson ML. Risk factors for adverse cutaneous reactions associated with intravenous vancomycin. J Antimicrob Chemother 1997; 39: 371–81. 10.1093/oxfordjournals.jac.a020861 9096187

[14] Levy M, Koren G, Dupuis L et al Vancomycin-induced red man syndrome. Pediatrics 1990; 86: 572–80. 10.1542/peds.86.4.572 2216623

[15] O’Sullivan TL, Ruffing MJ, Lamp KC et al Prospective evaluation of red man syndrome in patients receiving vancomycin. J Infect Dis 1993; 168: 773–6. 10.1093/infdis/168.3.773 8354921

[16] Wallace MR, Mascola JR, Oldfield EC III. Red man syndrome: incidence, etiology, and prophylaxis. J Infect Dis 1991; 164: 1180–5. 10.1093/infdis/164.6.1180 1955716

[17] Bauters T, Claus B, Schelstraete P et al Vancomycin-induced red man syndrome in pediatric oncology: still an issue? Int J Clin Pharm 2012; 34: 13–6. 10.1007/s11096-011-9593-z 22161495

[18] Wilson AP . Comparative safety of teicoplanin and vancomycin. Int J Antimicrob Agents 1998; 10: 143–52. 10.1016/S0924-8579(98)00025-9 9716291

[19] Bruniera FR, Ferreira FM, Saviolli LR et al The use of vancomycin with its therapeutic and adverse effects: a review. Eur Rev Med Pharmacol Sci 2015; 19: 694–700. 25753888

[20] Myers AL, Gaedigk A, Dai H et al Defining risk factors for red man syndrome in children and adults. Pediatr Infect Dis J 2012; 31: 464–8. 10.1097/INF.0b013e31824e10d7 22327873 PMC3333837

[21] Grant JM, Song WHC, Shajari S et al Safety of administering cefazolin versus other antibiotics in penicillin-allergic patients for surgical prophylaxis at a major Canadian teaching hospital. Surgery 2021; 170: 783–9. 10.1016/j.surg.2021.03.022 33894984

[22] Bratzler DW, Dellinger EP, Olsen KM et al Clinical practice guidelines for antimicrobial prophylaxis in surgery. Am J Health Syst Pharm 2013; 70: 195–283. 10.2146/ajhp120568 23327981

[23] WHO Guidelines Approved by the Guidelines Review Committee . Global Guidelines for the Prevention of Surgical Site Infection. World Health Organization, 2018.

[24] National Institute for Health and Care Excellence . Clinical Guidelines. Surgical Site Infections: Prevention and Treatment. National Institute for Health and Care Excellence (NICE), 2020. 31211539

[25] Honda H, Murakami S, Tokuda Y et al Critical national shortage of cefazolin in Japan: management strategies. Clin Infect Dis 2020; 71: 1783–9. 10.1093/cid/ciaa216 32133482

[26] Meiji Seika Pharma Co., Ltd . Vancomycin hydrochloride for intravenous infusion 0.5 g “MEIJI” [package insert in Japanese]. 2024. https://www.info.pmda.go.jp/go/pack/6113400A1219_1_07/?view=frame&style=XML&lang=ja.

[27] Japanese Society of Chemotherapy, Japan Society for Surgical Infection . Japanese Clinical Practice Guidelines for Antimicrobial Prophylaxis in Surgery [in Japanese]. 2016. https://www.chemotherapy.or.jp/uploads/files/guideline/jyutsugo_shiyou_jissen.pdf.

[28] Levy JH, Marty AT. Vancomycin and adverse drug reactions. Crit Care Med 1993; 21: 1107–8. 10.1097/00003246-199308000-00002 7687945

[29] Sivagnanam S, Deleu D. Red man syndrome. Crit Care 2003; 7: 119–20. 10.1186/cc1871 12720556 PMC270616

[30] Sahai J, Healy DP, Shelton MJ et al Comparison of vancomycin- and teicoplanin-induced histamine release and “red man syndrome”. Antimicrob Agents Chemother 1990; 34: 765–9. 10.1128/AAC.34.5.765 1694421 PMC171688

[31] Rybak MJ, Bailey EM, Warbasse LH. Absence of “red man syndrome” in patients being treated with vancomycin or high-dose teicoplanin. Antimicrob Agents Chemother 1992; 36: 1204–7. 10.1128/AAC.36.6.1204 1384423 PMC190318

[32] Renz CL, Laroche D, Thurn JD et al Tryptase levels are not increased during vancomycin-induced anaphylactoid reactions. Anesthesiology 1998; 89: 620–5. 10.1097/00000542-199809000-00010 9743397

[33] Konold VJL, Brothers AW, Kronman M et al Flushing an offensive term for vancomycin infusion reaction from the electronic medical record. Hosp Pediatr 2021; 11: e317–21. 10.1542/hpeds.2021-005993 34675085

